# A Vision Language-Based Framework for Detecting Industrial Mechanical, Electrical, and Plumbing Assets Using Unlabelled Data

**DOI:** 10.3390/s26082379

**Published:** 2026-04-12

**Authors:** Masoud Kamali, Behnam Atazadeh, Abbas Rajabifard, Yiqun Chen, Ensiyeh Javaherian Pour

**Affiliations:** The Centre for Spatial Data Infrastructures and Land Administration, Department of Infrastructure Engineering, The University of Melbourne, Melbourne, VIC 3010, Australia; behnam.atazadeh@unimelb.edu.au (B.A.); abbas.r@unimelb.edu.au (A.R.); yiqun.c@unimelb.edu.au (Y.C.); ensiyehj@student.unimelb.edu.au (E.J.P.)

**Keywords:** open-vocabulary object detection, vision language models, close-set object detectors, MEP assets

## Abstract

There have been significant advancements in object detection using extensive labelled datasets. However, existing learning-based approaches remain constrained in industrial environments, primarily due to the limited diversity in training datasets; the lack of generalisation of close-set detectors to unseen asset categories; and the inherent spatial and geometric complexity of mechanical, electrical, and plumbing (MEP) assets. To address this challenge, we propose a new approach that leverages pre-trained vision language models and close-set object detectors to detect unseen MEP assets using unlabelled data. Experimental results reveal the superior performance of Grounding DINO using Swin B transformer in open-vocabulary MEP asset detection, achieving the mean intersection over union (mIoU) of 0.6586 for valve detection and 0.4883 for pump detection. In addition, the combination of Grounding DINO (Swin B) and YOLOv8 outperforms other configurations in MEP asset detection, attaining the highest performance for both valve detection, with mean average precision at IoU = 0.5 (mAP50) of 0.928 and mean average precision over IoU threshold from 0.5 to 0.95 (mAP50:95) of 0.889, and pump detection, with corresponding values of 0.778 and 0.662, respectively. The quantitative and qualitative results of our approach were evaluated against fine-tuned Grounding DINO and fully supervised close-set object detectors.

## 1. Introduction

Industrial environments encompass a wide range of large and small-scale assets. The complexity of these assets surpasses that of residential buildings in terms of both geometry and scale. MEP assets play a critical role in numerous industrial plants, including petroleum refineries and water treatment facilities [1]. They provide essential services due to their functionality and features. In large-scale projects, such as healthcare or biotech industries, the cost of MEP assets can exceed 50% of the total investment [2]. Maintenance of these assets is necessary to enhance the efficiency of industrial plants [3]. The diversity of these assets in industrial plants is among the most critical factors to be considered at distinct phases of design, construction, and maintenance [4]. As a result, MEP assets necessitate more attention in terms of maintenance when compared to other building elements such as walls and doors. Efficient maintenance of MEP assets relies on accurately detecting and locating these assets within the complex industrial environment [5]. By leveraging object detection algorithms, industrial plant operators can streamline maintenance activities and improve asset monitoring [6].

Object detection is a prominent task in computer vision and scene understanding. This task involves the classification and localisation of objects in 2D/3D datasets, such as images and point clouds. Object detection has various applications, including healthcare [7], autonomous driving [8], construction [9], and disaster management [10]. With the emergence of artificial intelligence (AI), object detection and scene understanding have seen a remarkable transformation across various domains [11]. However, learning-based object detection algorithms require large-scale labelled dataset to extract and learn patterns from images [12]. Labelling a sufficient amount of data for each object is a tedious and labour-intensive task [13]. This problem is more challenging for complex objects, such as industrial assets, due to the complexities in their geometries, contextual variations, and accessibility [14]. Additionally, current benchmark datasets face limitations in their class diversity [15], as most of them do not contain complex industrial assets. Accordingly, close-set detection methods cannot be directly employed to detect the location of unseen objects [16].

To address the limitations of close-set approaches, several studies have focused on zero-shot or open-vocabulary detection [17,18,19]. In contrast to close-set methods, open-vocabulary object detection refers to detection of unseen objects that are not covered by pre-defined classes. Leveraging semantic information, such as textual data, in object detectors models enables them to learn rich representations of both images and their corresponding texts. Utilising vision language models for open-vocabulary object detection has resulted in significant improvements in detecting unseen objects [16]. The vision language models extract semantic relationships between image–text pairs and allows the generalisation of visual features to unseen classes.

In this paper, we propose using vision language models to annotate unlabelled MEP assets in industrial scene images, followed by training a close-set object detector on the generated labelled dataset. The use of vision language model eliminates the need for manual labelling of MEP assets in images. Additionally, by leveraging the superior performance of close-set object detectors in object detection compared to open-vocabulary methods [20], the close-set object detector is trained using the dataset generated by the vision language model. We employ state-of-the-art (SOTA) vision language models to assess the results in open-vocabulary MEP asset detection within industrial scene images. In this study, Grounding DINO [20], GroundedSAM [20,21], Detic [15], OWL-ViT [22], and OWLv2 [23] are utilised to detect unseen classes of MEP assets and annotate unlabelled images. Subsequently, the annotated dataset is used to train close-set object detectors, including YOLOv8 [24], YOLO-NAS [25], and Detr [26].

The primary contributions of this research can be summarised as follows:

1. The proposed approach represents the first study to utilise vision language models to annotate unseen MEP assets in industrial scene images, consequently eliminating the need for extensive manual labelling of these assets for object detection.
2. The combination of SOTA vision language models, including Grounding DINO, GroundedSAM, OWLv2, OWL-ViT, and Detic, along with close-set object detectors such as YOLOv8, YOLO-NAS, and Detr, are employed for MEP asset detection.

The remainder of the paper is structured as follows: The second section explores current image-based object detection approaches in industrial contexts and SOTA open-vocabulary object detection methods. The third section presents the proposed approach for MEP asset detection using unlabelled image datasets. The results and experiments are detailed in the fourth section. The fifth section also discusses the results and limitations. The last section provides the main findings of this study and outlines future directions.

## 2. Literature Review

In the industrial landscape, object detection plays a critical role across various applications, including infrastructure maintenance [27,28,29,30], defect detection [31,32,33], and construction monitoring [34,35,36]. The research literature has extensively examined datasets utilised for image-based object detection in industrial context [27,28,37]. Following that, semi-supervised and zero-shot methods have been explored to reduce the necessity of labelled training dataset [38,39,40,41]. Then, pre-trained vision language models have been investigated for open-vocabulary object detection [42,43].

### 2.1. Image-Based Object Detection in Industrial Context

To detect objects in industrial environments, a variety of datasets have been utilised in prior studies [27,28,29]. Zhang et al. [27] used a database comprising 4800 images of manually labelled cracked and non-cracked concrete bridge decks. Lu et al. collected images from the construction site using a drone, and a total of 1516 samples were labelled for training. He et al. [28] curated the UAV Near–Far Scene Images (UNFSI), incorporating the Crack500 and CFD public datasets along with road crack images captured by both mobile phones and UAVs. Wang and Su [29] utilised the COCO-Bridgeþ2021 dataset derived from real bridge inspection reports. The dataset involved extracting bearing bounding box coordinates from ground truth labels and cropping the corresponding areas in original images. Chu [44] utilised the CrackLS315 dataset for training, fine-tuned on 50 HR crack images from the Yinpenling Bridge. Yang et al. [45] used a dataset included 1969 images with 4385 annotations, covering spalling, exposed rebar, and efflorescence as common defect types. Zhao et al. [30] employed a dataset consisting of 2500 images, each with a pixel resolution of 640 × 640 to detect and locate concrete dam damages in 3D reconstruction models. Yan et al. [46] created an instance-level segmentation dataset for object detection and segmentation tasks within industrial environments. Ding et al. [47] used a dataset comprised 2800 images capturing cracks, with 1850 sourced from public datasets, and an additional 950 images, featuring some blurring, were collected by UAVs. Ye et al. [33] used a dataset of 1000 collected fastener defect images.

#### MEP Asset Detection

Kufuor et al. [48] proposed a deep learning framework to detect MEP components such as sockets, switches, and radiators in 360° and phone images. A Faster R-CNN model was fine-tuned via transfer learning for component localisation across diverse formats. Wei et al. [49] employed a camera imaging model to align on-site photographs with BIM projections, utilising the Segment Anything Model (SAM) for pipeline segmentation. Wang and Fang [50] developed an automated BIM reconstruction method based on 2D engineering drawings, in which MEP elements were identified using a semantic segmentation model trained on a custom dataset. Component coordinates and dimensions were then extracted through contour analysis and bounding box detection. Edwards et al. [51] introduced an auto-linking technique that integrated 3D scan data with asset management systems by leveraging object detection and optical character recognition (OCR) to extract semantic information from images. The approach was trained on a labelled piping and instrumentation diagrams (P&IDs) dataset to detect labels, symbols, and connections. Guerra et al. [52] proposed a synthetic data generation approach tailored for industrial pipeline detection. They employed AlexNet [53] and FCN [54] for object detection and semantic segmentation on the generated image dataset. Guo et al. [55] employed Fast R-CNN [56] to detect tube-like features by identifying edges in the images. Seed points were then randomly placed to facilitate the reconstruction of tubes within multi-pipe structures. Bhanbhro et al. [57] investigated symbol recognition within 2D engineering drawings. This approach utilised YOLO and Faster R-CNN [56] models to detect industrial symbols in P&IDs. Guo et al. [31] captured 7000 images depicting diverse types of pipe defects using the Pipeline Capsule Machine (PCM). While several large-scale benchmark labelled datasets are available for industrial environments [58,59,60], the detection of most industrial assets, including MEP assets, still relies heavily on manually labelled training data, limiting scalability and generalisation to unseen asset categories. To address this limitation, there are several methods, such as semi-supervised learning methods employed to reduce the annotation workload and leverage both labelled and unlabelled data [31].

### 2.2. Semi-Supervised Learning-Based Methods

Semi-supervised methods leverage both labelled and unlabelled data, reducing annotation workload while efficiently training algorithms [61]. Lin et al. [38] integrated self-supervised learning and transformers. The incorporation of a visual transformer (ViT) and a facial recognition-like framework facilitated efficient distress detection, addressing challenges associated with limited labelled data. Gopalakrishnan et al. [62] employed a pre-trained deep convolutional neural network (DCNN) with transfer learning for automated pavement crack detection. The approach addressed the limitation of scarce labelled data by leveraging pre-trained models. A total of 1056 labelled pavement images were used to evaluate the performance of the pre-trained DCNNs. Gupta et al. [39] presented an approach for P&ID symbol detection using a semi-supervised method trained on a large dataset to enhance generalisation across diverse P&ID styles and symbolical representations. Shamsabadi [63] combined supervised training, semi-supervised consistency regularisation, and self-training with certainty-based pseudo-labelling. The results indicated that by utilising only 2% of the total labelled data, the mIoU was merely 2.6% and 4.7% lower for concrete and asphalt, respectively, compared to the best-performing model trained on 100% of labelled data. However, semi-supervised learning methods still require labelled data and therefore do not fully remove the dependency on manual annotation, limiting their applicability to detecting unseen asset classes. Some studies have explored zero-shot detection approaches to reduce the reliance on labelled training data and enable the detection of unseen classes in industrial settings.

### 2.3. Zero-Shot Learning-Based Methods

Zero-shot learning is a machine learning technique that enables models to recognise objects or concepts they have never seen during training [64]. Hong et al. [65] investigated advanced domain adaptation methods for improving the generalisation of semantic segmentation models in construction site monitoring. They introduced a novel approach addressing the homogeneous domain adaptation problem, involving target-specific copy–paste data augmentation, prototypical pseudo-label denoising, target structure learning, and knowledge distillation. Feng and Zhao [40] introduced a zero-sample fault diagnosis approach through a novel fault description-based attribute transfer method. Zhuo and Ge [66] proposed a generative approach using generative adversarial networks to tackle the any-shot learning problem in fault diagnosis. However, FAGAN’s performance relied on accurate fault attributes; additionally, if a fault is not represented in the attribute space, FAGAN cannot synthesise samples for that specific fault. Li et al. [41] introduced the Zero-Shot Class Knowledge Graph (ZS-CKG) method for real-world surface defect detection. The approach involved constructing a class knowledge graph to establish relationships between base and novel defect classes and utilised a graph convolutional neural network to learn class features. Azeem and Zaidi [67] introduced signature attributes for the dataset, fine-tuned a CNN architecture, and applied zero-shot learning using the Class and Image Embedding (CVIE) approach. Notably, increasing the number of signature attributes led to a loss in zero-shot accuracy, with better results achieved using a smaller set of attributes. Di Maggio et al. [68] employed a GAN with a cycle consistency loss function, known as cycleGAN, to generate synthetic data. The generative model was trained to convert wavelet images of simulated vibrational signals into authentic data obtained from machinery. Subsequently, three CNN-based diagnosis models were trained using the transfer learning technique, incorporating real, simulated, or synthetic data related to damaged machinery [68]. Although various methods, including GANs, transfer learning, and knowledge graph have been investigated for zero-shot detection, their effectiveness remains constrained by reliance on predefined attributes and domain-specific representations. This reliance limits their ability to represent the complex geometry and contextual variability of industrial MEP assets. In contrast, open-vocabulary object detection models can capture complex relationships between assets through the combination of language and vision. In this study, we use the term “open-vocabulary object detection” instead of “zero-shot learning”, highlighting the integration of language and visual information within vision language models.

### 2.4. Vision Language Models for Object Detection

Zhao et al. [42] proposed a method to leverage vision and language models to generate pseudo-labels for object detection in unlabelled images. They employed vision and language models to categorise each region of an image into object categories required for downstream tasks. Bravo et al. [69] proposed a method using an image–caption pairs dataset, which learned to detect novel object classes alongside a given set of known classes. The approach involved a two-stage training process. Initially, a location-guided image–caption matching technique was employed to weakly supervise the learning of class labels for both novel and known classes. Tan et al. explored the feasibility of large language models for data annotation [70]. Gu et al. [43] introduced a technique wherein knowledge was extracted from a pre-trained open-vocabulary image classification model and incorporated into a two-stage detector. Xie and Zheng [71] introduced a method to align vision language embeddings to transfer the generalisation capabilities of CLIP to a one-stage object detector, specifically YOLOv5. Cheng et al. [72] proposed YOLO-World, an innovative approach that enhances the YOLO object detection series by incorporating open-vocabulary detection capabilities. They employed a novel Re-parameterizable Vision Language Path Aggregation Network (RepVL-PAN) and a region-text contrastive loss to facilitate effective interactions between visual and linguistic information. While these studies focus on general use cases, they do not specifically address MEP asset detection in industrial environments. One of the few notable studies in this domain is Omni-Scan2BIM [73], which demonstrated the use of a pre-trained vision foundation model to detect MEP-related assets within industrial scenes. This study integrated a generic feature extractor (DINOv2 [74]) with SAM to adopt a single-shot approach that relies on a sample image and its corresponding reference mask for MEP asset detection. In contrast, our approach focuses on zero-shot detection without requiring sample images and reference masks. While Omni-Scan2BIM utilised only DINOv2 for feature extraction, we evaluated multiple SOTA vision language models to explore their effectiveness for open-vocabulary MEP asset detection. Moreover, our study evaluates MEP assets with more complex geometry, such as valves and pumps, to demonstrate the generalisation capability of vision language models in a wider range of industrial environments. Based on the literature and diverse nature of industrial assets within complex scenes, the utilisation of vision language models for open-vocabulary MEP asset object detection in industrial settings is still overlooked.

## 3. Methodology

**Figure 1:**
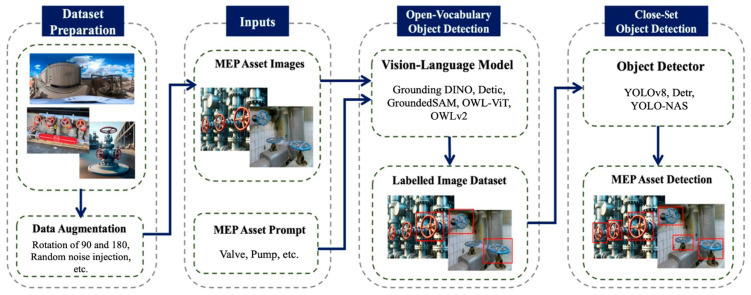
The proposed MEP asset detection approach.

Due to the diversity and geometrical complexity of MEP assets in industrial environments, a substantial amount of the labelled training dataset is required to train close-set object detectors. The process of annotating images is known to be a time-consuming and expensive task [31]. Consequently, adopting a zero-shot annotation approach can significantly mitigate the cost and time of dataset preparation. Figure 1 illustrates the proposed approach, which uses vision language models as open-vocabulary object detectors to annotate unlabelled image datasets. The inputs consist of unlabelled images of MEP assets and corresponding text prompts containing the name of MEP assets. The pre-trained vision language models are then employed for open-vocabulary object detection. In this phase, MEP assets are detected in the images using provided text prompts, and the images are then annotated. The annotated images serve as the training dataset for close-set object detectors. Given the superior performance of close-set object detectors compared to open-vocabulary methods [20], we trained close-set object detectors to learn and extract patterns from annotated data and detect MEP assets.

**Phase 1: Dataset Preparation**

**Figure 2:**
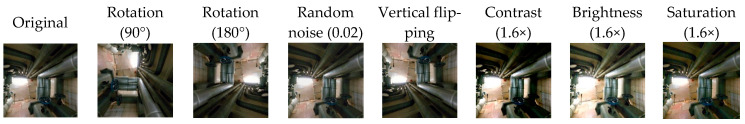
Augmentation techniques for image dataset.

In this study, we used unlabelled images from industrial scenes in three categories: (1) real flat images from industrial scenes; (2) real spherical panorama images; and (3) images produced by generative AI. Since panorama images encompassed a significant number of assets, each panorama image was split into six overlapping flat images using Meshroom software (version 2025.1.0) [75]. In addition, to generate complex industrial scenes and diverse types of MEP assets, generative AI was employed to create images. In this study, a large-scale vision language model [76] was utilised to describe MEP assets in industrial settings and generate captions. Subsequently, a text-to-image generation model [77] was employed to generate new images based on the captions. Prompt engineering was used to produce near-realistic images of industrial assets and environments. A total of 262 flat images, 32 spherical panorama images, and 35 images produced by generative AI were employed for data augmentation. To augment the image dataset, image processing methods have been utilised. According to the complexity of assets in industrial environments, rotations of 90° and 180°; vertical flipping; and random noise injection, as well as adjustments in contrast, brightness, and saturation were applied to expand the image dataset. Ultimately, 2610 images were selected for open-vocabulary object detection using vision language models. Figure 2 shows the augmentation techniques that have been used in this research.

To ensure a robust experimental protocol, the dataset was partitioned at the level of original images prior to augmentation and pseudo-label generation, thereby preventing data leakage between training and evaluation subsets. All augmented samples derived from a given image were assigned to the same subset. Likewise, all perspective views generated from a single spherical panorama were assigned to a single subset to avoid overlapping scene content. Furthermore, the images created using generative AI were excluded from the test set to ensure that evaluation was conducted exclusively on real-world industrial images.

**Phase 2: Open-Vocabulary Object Detection**

The limitations of close-set object detectors lie in their constrained ability to handle novel or unseen classes. Open-vocabulary object detection emerges as pivotal in addressing this limitation, offering the flexibility to detect a broader spectrum of objects without the need for predefined classes.

Vision language models are instrumental in open-vocabulary object detection due to their ability to bridge the semantic gap between textual descriptions and visual content. They generalise the rich contextual information embedded in textual prompts to detect objects that have not been labelled during training. Pre-trained vision language models capture intricate features and relationships between images and their textual descriptions due to the large-scale training datasets. In this study, we used pre-trained vision language models to annotate images based on textual prompts, eliminating the need for extensive manual labelling of unseen MEP asset classes. The SOTA pre-trained vision language models employed in this research include Grounding DINO, GroundedSAM, Detic, OWL-ViT, and OWLv2.

Grounding DINO: Grounding DINO is an open-set object detection model that integrates the DINO architecture with grounded pre-training. It is designed to detect arbitrary objects specified by language inputs, enabling generalisation to unseen objects. The model seamlessly fuses language and vision modalities, which includes a feature enhancer, a language-guided query selection, and a cross-modality decoder. The Grounding DINO model utilised the Swin [78] backbone pre-trained weights. The Swin B (base) backbone comprises 145M parameters and has been trained with 22k classes, while Swin T (tiny) consists of 86M parameters and was trained with 1k classes.

Grounded SAM: This model combines Grounding DINO with the Segment Anything Model (SAM) to detect unseen objects and then segment them in images.

Detic: Detic is a method for open-vocabulary object detection that uses image-level supervision. It decouples localization and classification in object detection and focuses on training the classifier using image-level labels. Detic demonstrates the ability to train a detector with all 21,000 classes of the ImageNet dataset and generalise to new datasets without fine-tuning.

OWL-ViT: Vision transformer for open-world localisation (OWL-ViT) is a model designed for open-vocabulary object detection. It integrates image and text embeddings to enable zero-shot generalisation, allowing the model to detect objects that it has not seen during training. OWL-ViT employs a contrastive vision language pre-training approach, where images and text are embedded into a shared space. Subsequently, the model transfers these pre-trained encoders to object detection by eliminating token pooling and directly connecting lightweight object classification and localisation heads to the image encoder output tokens.

OWLv2: An open-vocabulary object detection model that utilises a self-training approach, where the model is trained on a large dataset using pre-training techniques like CLIP [79]. Open-vocabulary object detection is enabled by replacing the fixed classification layer weights with the class name embeddings obtained from the text model.

In this study, we used pre-trained vision language models to train close-set object detectors [80]. Notably, the MEP asset classes were not included in the original training dataset of the vision language models, ensuring the generalisability of the approach to previously unseen categories. The unlabelled dataset of MEP assets and text prompts were utilised to detect unseen assets through pre-trained vision language foundation models. Then, close-set object detectors were trained using generated labelled image datasets. Ultimately, the trained model can detect MEP assets in industrial settings.

**Phase 3: Close-Set Object Detection**

Upon MEP asset detection using vision language models, the next step involved the preparation of a training dataset format to facilitate the training of close-set object detectors. Training dataset formats are structures that define how data should be organised and annotated to train learning-based algorithms, including object detectors. The two formats used in this research were COCO (common objects in context) and YOLO (you only look once).

To evaluate the performance of MEP asset detection using different object detection architectures, we utilised the annotated dataset to train YOLOv8, YOLO-NAS, and Detr. YOLOv8 and YOLO-NAS are built on convolutional neural networks (CNNs), incorporating advanced backbone and head designs to improve spatial feature extraction and object localisation. In contrast, Detr employs a transformer-based architecture that formulates object detection as a direct set prediction task. It uses a self-attention mechanism to model global relationships within the image to eliminate the need for components such as anchor boxes and non-maximum suppression. This architectural diversity allows for a comparative assessment of CNN-based and transformer-based detection paradigms for MEP asset detection.

## 4. Results

The efficacy of the proposed approach is evaluated through the detection of MEP assets, including valves and pumps within industrial settings. Valves are essential for maintaining operational efficiency and preventing disruptions, inefficiencies, and safety risks in industrial processes [81,82]. In addition, pumps play a critical role in fluid transport systems, where failures can result in significant downtime and increased maintenance costs [83].

To evaluate detection performance, standard metrics were employed, including mIoU and mAP. The mIoU represented the average overlap between the predicted and ground truth bounding boxes across all N detections, defined as:

$$mIoU=\frac{1}{N}\sum_{i=1}^{N}\frac{\left|B_{p}^{\left(i\right)}\;\cap \;B_{gt}^{\left(i\right)}\right|}{\left|B_{p}^{\left(i\right)}\;\cup \;B_{gt}^{\left(i\right)}\right|}$$

where $B_{p}^{\left(i\right)}$ and $B_{gt}^{\left(i\right)}$ denote the predicted and ground truth bounding boxes for the i-th instance, and ∣⋅∣ represents the area of intersection and union. This metric provided an overall indication of localisation accuracy based on spatial agreement between predictions and ground truth instances.

The mAP was derived from the average precision, which was calculated as the area under the precision–recall curve:

$$AP=\int_{0}^{1}p\left(r\right)\;dr$$

where p(r) denotes precision as a function of recall r. The mAP was then obtained as the mean AP across all C object classes:

$$mAP=\frac{1}{C}\sum_{c=1}^{C}{AP}_{c}$$

where $AP_{c}$ represents the average precision for class c. In this study, mAP50 referred to AP evaluated at an IoU threshold of 0.5, while mAP50:95 was obtained by averaging AP over multiple IoU thresholds ranging from 0.5 to 0.95 with a step size of 0.05:

$${mAP}_{50:95}=\frac{1}{T}\sum_{t\in \{0.5,\;0.55,\;\ldots ,0.95\}}{AP}_{t}$$

where T denotes the number of thresholds and $AP_{t}$ represents the average precision at threshold t. These metrics enabled a comprehensive evaluation by assessing detection performance across both lenient and strict localisation requirements. Section 4.1 discusses the quantitative results achieved using open-vocabulary object detection and MEP asset detection. In Section 4.2, we illustrate the qualitative results regarding the combination of approaches employed for MEP asset detection.

### 4.1. Quantitative Results

In this section, we discuss the outcomes of the open-vocabulary MEP asset detection using pre-trained vision language models and leveraging close-set object detectors (Section 4.1.1). Our analyses include the results of training classification loss for MEP asset detection (Section 4.1.2). We have evaluated SOTA vision language models and close-set object detectors such as YOLOv8 and YOLO-NAS, along with a Grounding DINO fine-tuned model and fully supervised methods. Following this, Section 4.1.3 presents the numerical results of the proposed networks, highlighting metrics such as mAP50 as well as mAP50:95.

#### 4.1.1. Open-Vocabulary MEP Asset Detection Using Vision Language Models

In this section, we evaluated the results of six pre-trained vision language models to detect industrial valves and pumps in an open-vocabulary MEP asset detection task. Using consistent text prompts, we tested these models on the image dataset provided in Phase 1: Dataset preparation. The final dataset comprising 2610 images was distributed across valve and pump detection tasks, with each task further divided into training, validation, and test subsets, ensuring no overlap between subsets. Among the 2610 augmented images, 1327 were used for the valve detection task, while the remaining 1283 images were employed for the evaluation of pump detection. For each asset class, the dataset was partitioned into training, validation, and test subsets using an 80%/10%/10% split, providing sufficient data for model training while maintaining an independent evaluation set. All experiments were conducted exclusively on real industrial images, ensuring that the test data reflects real-world conditions and that the reported performance was not affected by synthetic data characteristics. The dataset partitioning strategy described in Section 3 ensured that no augmented or synthetic samples were shared between the training and evaluation sets. To compare the results, we manually labelled the image dataset and calculated the mIoU based on ground truth labels. Table 1 presents the mIoU results for each vision language model. Remarkably, Grounding DINO (Swin B) demonstrates superior performance compared to other pre-trained models, achieving a mIoU of 0.6586 for valve detection and 0.4883 for pump detection. In contrast, OWL-ViT model displays a lower mIoU result of 0.0217 for valves and 0.0519 for pumps within industrial scenes.

Table 2 depicts the differences in mIoU obtained through the combination of vision language models and close-set object detectors, relative to their performance without integration. YOLOv8 and YOLO-NAS were trained as close-set object detectors using the annotated dataset generated through vision language models. YOLOv8 consistently produced slight improvements in mIoU compared to open-vocabulary object detection (Table 1), indicating stable performance across different model combinations. As shown in Table 2, consistent positive differences were observed across both valve and pump detection tasks, including +0.0027 and +0.0031 for Grounding DINO (Swin B). Detic and OWLv2 exhibited comparatively larger gains, with improvements of +0.0509 and +0.0492, respectively. In contrast, the integration of YOLO-NAS with Grounding DINO resulted in a reduced mIoU across its configurations, indicating less consistent performance.

#### 4.1.2. Training Classification Loss

**Figure 3:**
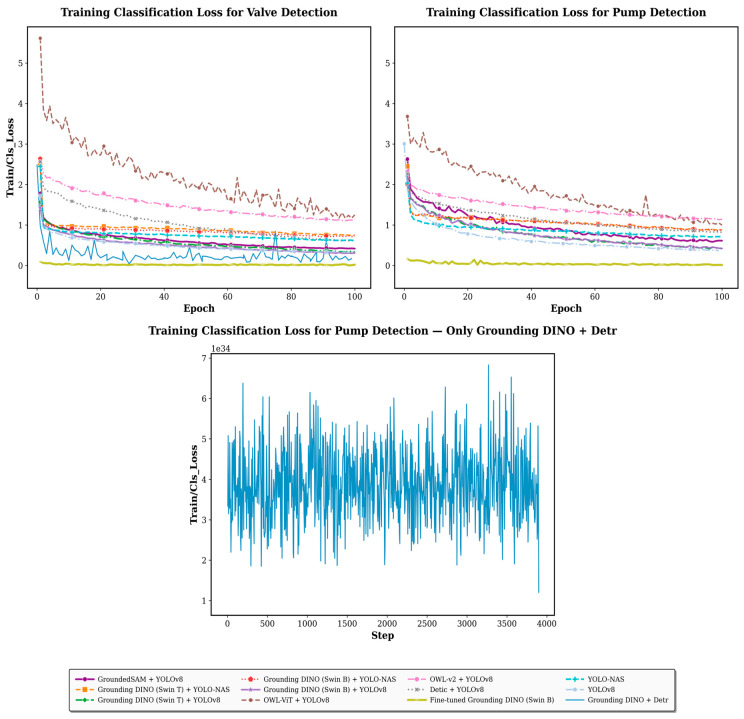
Training classification loss of vision language models and close-set object detectors for valve detection (**left**) and pump detection (**right**). The (**bottom**) plot presents the training classification loss for pump detection using Grounding DINO and Detr, highlighting the fluctuation pattern of the classification loss in the Detr object detector.

In the context of object detection, the training classification loss represents the error or discrepancy between the predicted class labels and the actual ground truth labels for the objects in the training dataset. Figure 3 shows the results of this metric over 100 epochs for valve detection (top-left), pump detection (top-right), and a distinct combination using Grounding DINO (Swin B) and Detr for pump detection (bottom). As depicted, OWL-ViT and YOLOv8 exhibits higher classification loss, while the combination of Grounding DINO (Swin B) and YOLOv8 demonstrates lower classification loss compared to other combination approaches. YOLOv8 outperforms YOLO-NAS in training classification performance when both are applied to the same datasets generated by Grounding DINO (Swin B and Swin T) and the fully supervised methods. The fine-tuned Grounding DINO (Swin B) model achieves the lowest classification loss, surpassing both the fully supervised and combination approaches.

While the combination of Grounding DINO (Swin B) with YOLOv8 and YOLO-NAS converged steadily in both valve and pump detection, the integration of Grounding DINO with Detr exhibits high-frequency fluctuations in training classification loss across both object types, particularly in the pump detection task. This behaviour suggests that Detr struggles to consistently learn and generalise patterns from the training dataset.

#### 4.1.3. mAP Results

In this study, we evaluated the performance of object detection approaches using mAP metrics at different confidence thresholds. Table 3 depicts the results for valve and pump detection across various models. Among the combination approaches, Grounding DINO (Swin B) with YOLOv8 achieved the highest performance for valve detection (mAP50: 0.928, mAP50:95: 0.889) and yielded strong results for pump detection (mAP50: 0.778, mAP50:95: 0.662), highlighting the effectiveness of this combination across both asset types. Although the fine-tuned Grounding DINO model showed a slight improvement in valve detection, with an increase of 0.058 in mAP50 and 0.075 in mAP50:95, the combination approach remains highly competitive without relying on labelled training data.

For valve detection, the fully supervised YOLOv8 model achieved the highest mAP50 of 0.9917, whereas the fine-tuned Grounding DINO (Swin B) model attained the highest mAP50:95 of 0.964. For pump detection, the fine-tuned Grounding DINO (Swin B) model outperformed all other methods, achieving the highest overall scores with mAP50 of 0.937 and mAP50:95 of 0.755. Models utilising the Swin T and Swin B transformer backbones, such as Grounding DINO, consistently demonstrated strong performance across both valve and pump detection tasks, whereas OWL-ViT and OWLv2 exhibited substantially lower accuracy, particularly in terms of mAP50:95.

### 4.2. Qualitative Results

**Figure 4:**
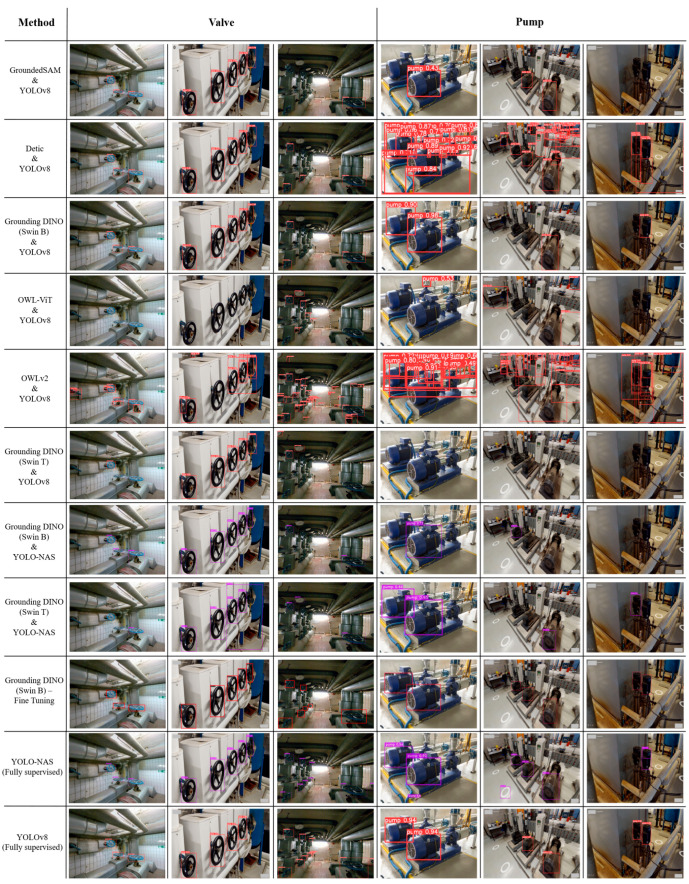
The visualisation outputs of MEP asset detection using vision language models and close-set object detectors. Left and right images collected from [84]. The middle image for valve and pump detection, as well as the rightmost image for pump detection, were captured from a building in Melbourne, Australia.

To explore the qualitative results of MEP asset detection, we thoroughly examined the proposed approach using real images. This section aims to demonstrate the robustness and effectiveness of proposed approach for MEP asset detection. Figure 4 displays the outcomes achieved through the combination of vision language models and close-set object detectors for valve and pump detection. We also compared the results with fully supervised and fine-tuned models of Grounding DINO (Swin B).

In the first image, all three valves were detected by GroundedSAM and YOLOv8; Detic and YOLOv8; Grounding DINO (Swin B) and YOLOv8; and Grounding DINO (Swin B) and YOLO-NAS. Conversely, Grounding DINO (Swin T) with YOLO-NAS and YOLOv8 only detected one of the three valves, and OWL-ViT failed to detect any valves in the image. In the second image, GroundedSAM, Detic, and Grounding DINO (Swin B and T) combined with YOLOv8, as well as Grounding DINO (Swin B) with YOLO-NAS successfully detected all valves. However, the fully supervised YOLOv8 method failed to identify the furthest valve in this image; additionally, OWLv2 produced false positives, indicating reduced precision in distinguishing valve instances. In the third image, Detic and YOLOv8 detected four valves, surpassing the other combination methods in detection accuracy. Grounding DINO (Swin B) and YOLOv8 and Grounding DINO (Swin B) and YOLO-NAS detected three and two valves, respectively. In addition, Grounding DINO (Swin T) combined with YOLOv8 or YOLO-NAS exhibited false positives by misclassifying non-valve objects as valves. In the third image, smaller valves were solely detected in the fine-tuned model of Grounding DINO and the fully supervised approaches.

The pump detection results revealed substantial variations in performance across different model combinations. Both Detic and OWLv2, when integrated with YOLOv8, yielded false positives, indicating their inability to learn the distinctive geometric and contextual features of pump assets. To evaluate the generalisation capability of the proposed approach, both horizontal and vertical pump types were examined. In the last image, Grounding DINO (Swin B) combined with YOLOv8, as well as both Swin B and Swin T variants paired with YOLO-NAS, successfully detected one of two vertical pumps. Notably, the fine-tuned version of Grounding DINO (Swin B) demonstrated superior performance, outperforming the fully supervised YOLOv8 and YOLO-NAS models in detecting pump assets.

## 5. Discussion

The results demonstrated the effectiveness of open-vocabulary MEP asset detection by leveraging the combination of vision language models and close-set object detectors. We selected images from industrial scenes containing multiple assets to assess the efficacy of the proposed approaches, as detecting specific assets amongst others in complex industrial scenes is more challenging than in images with single assets and no background. Utilising the linguistic capabilities of vision language models enhanced our understanding of complex environments such as industrial contexts. While we employed the same dataset and text prompts for open-vocabulary object detection, Table 1 shows that Grounding DINO (Swin B) achieved a higher mIoU compared to the other vision language models. Additionally, Table 2 demonstrates that the mIoU results improved when utilising YOLOv8 with the generated labelled dataset. Moreover, in some cases, the combination of vision language models and close-set object detectors led to achieving lower training classification losses in valve and pump detection compared to fully supervised models using the same dataset. For instance, both Grounding DINO (Swin B or Swin T) and YOLOv8 exhibited a lower training classification loss than YOLO-NAS fully supervised models after 100 epochs. Nonetheless, based on the qualitative results, fully supervised models like YOLO-NAS and YOLOv8 detected more true positive valves than the proposed approach. For instance, in the third image of Figure 4, only the fully supervised methods and the fine-tuned Grounding DINO model successfully detected industrial valves located at greater distances from the camera and with smaller dimensions.

Table 4 presents the precision, recall, and F1-scores obtained by combining vision language models with close-set object detectors for valve and pump detection. The results demonstrate that the proposed pseudo-labelling pipeline produced reliable supervisory signals for downstream training, with Grounding DINO (Swin B) consistently achieving the highest performance among the evaluated vision language models. The integration with YOLOv8 demonstrated strong detection performance, achieving F1-scores of 0.89 for valves and 0.764 for pumps, closely approaching the fully supervised models. The fine-tuned Grounding DINO further reduced this gap, attaining F1-scores of 0.965 and 0.92 for valve and pump detection, respectively. In contrast, the open-vocabulary models such as OWL-ViT and OWLv2 exhibit lower precision and recall, indicating increased false positives and missed detections in complex industrial scenes. Valve detection consistently outperformed pump detection across all configurations, highlighting the greater challenges posed by occlusion, scale variation, and visual complexity. While the fully supervised models achieved the highest overall performance, these results demonstrate that the proposed approach remains a competitive alternative with reduced dependence on manual annotation. However, its performance is inherently dependent on the quality of pseudo-labels generated by vision language models. Despite the findings indicating that these labels provide effective supervision, errors such as false positives, missed detections, and imprecise localisation may be incorporated into the training process. This limitation is particularly evident in complex industrial scenes characterised by occlusions and visually similar components.

The inference time results presented in Table 5 highlight notable differences in computational efficiency across the evaluated vision language models, primarily driven by variations in architectural complexity and processing pipelines. OWL-ViT achieved the fastest inference due to its relatively lightweight detection framework. In contrast, Grounding DINO introduced additional computational overhead through cross-modal feature interactions, resulting in moderately increased inference time. The models with increased capacity or complex detection pipelines, such as Detic and Grounding DINO (Swin B), exhibited a reduced throughput. GroundedSAM introduced additional latency due to its sequential segmentation stage following detection. OWLv2 recorded the longest inference time, reflecting its more computationally intensive inference design with multi-scale ensemble inference.

### Limitations

Employing pre-trained vision language models for open-vocabulary object detection involves two inputs: text prompts and images. Prompt engineering thus plays a critical role in open-vocabulary object detection. Additionally, the diversity of MEP assets and the spatial complexity of industrial scene images directly impact the accuracy of object detection. The challenges and limitations that vision language models face in detecting MEP assets include:

- **Prompt Engineering**: Vision language models utilise various prompt engineering techniques to enhance their understanding of visual features [85], such as task instruction [86], retrieval-based [87], and chain-of-thought prompting [88]. In this study, we used text prompts in two distinct steps, including (1) creating images using generative AI; and (2) object detection with vision language models. To generate images, a large-scale vision language model was utilised to describe MEP assets in industrial scene images and create captions. We then modified the caption of each image to generate new images, considering criteria such as the size of assets, colours, quantity, asset relationships, and background scenes. Subsequently, a text-to-image generation model was employed to generate new images from the modified captions and to blend multiple real or synthetic images, enabling the creation of new industrial scene images. For the purpose of object detection, we employed consistent text prompts across all vision language models, including the name of the MEP asset, such as “valve” and “pump”. However, while using different text prompt techniques for each image to describe industrial scene enhances the accuracy of object detection, this approach cannot be generalised across different vision language models and input images.
- **Diversity of Industrial Assets**: Industrial environments encompass a wide range of MEP assets, each characterised by its unique geometry and semantic. This diversity presents a significant challenge for open-vocabulary object detection. For instance, detecting diverse types of valves and pumps in each industrial context requires expertise and extra information. The complex geometries of assets like pumps and motors can reduce the accuracy of object detection. Additionally, the colour and size of each MEP asset may vary across different industrial scenes. The complexity of industrial scenes poses considerable challenges for open-vocabulary object detection. MEP assets, such as electrical conduit and plumbing pipes, with similar geometry can result in false detections. Moreover, image resolution is pivotal for accurate object detection, as it directly influences feature extraction from the labelled training dataset in close-set object detectors. The evaluation in this study was limited to two representative MEP asset classes, which may constrain the generalisation of the proposed approach to a broader range of asset types.

## 6. Conclusions

This study proposed an approach to detect MEP assets in industrial environments using unlabelled image datasets. The pre-trained vision language models were utilised to detect unseen classes of MEP assets and annotate unlabelled industrial scene images. The generated labelled dataset was then employed to train close-set object detectors and then detect MEP assets in images. The proposed approach demonstrated the capability to generalise across diverse MEP assets within complex industrial scenes. The findings indicated that Grounding DINO model outperformed the other pre-trained vision language models in open-vocabulary MEP asset detection. In addition, the Swin B transformer achieved better results than Swin T when applied as the backbone in the Grounding DINO model. The combination of YOLOv8 with vision language models enhanced the mIoU results compared to open-vocabulary MEP asset detection using vision language models alone. Overall, the results revealed that the proposed framework provides a competitive alternative to fully supervised approaches while reducing the reliance on labelled datasets.

Furthermore, future research directions include incorporating spatial relationships between MEP assets to improve contextual understanding and detection accuracy in complex industrial environments. Retrieval-augmented generation (RAG) can be explored to integrate external knowledge sources, such as technical specifications and engineering documents, to enhance semantic understanding of assets. In addition, learnable prompts can be developed to dynamically adapt textual queries based on specific industrial contexts, while leveraging additional information such as asset attributes and operational details to further improve open-vocabulary object detection performance.

## Figures and Tables

**Table 1:** The mIoU metric for open-vocabulary object detection using vision language models.

Asset\VLM	Grounding DINO (Swin B)	Grounding DINO (Swin T)	GroundedSAM (Swin B)	Detic	OWL-ViT	OWLv2
Valve	0.6586	0.6127	0.1721	0.3559	0.0217	0.278
Pump	0.4883	0.424	0.1103	0.3131	0.0519	0.2191

**Table 2:** The mIoU result differences for valve and pump asset detection between the independent use vision language models and their integration with close-set object detectors.

		Asset	
Vision Language Model	Close-Set Object Detector	Valve	Pump
Grounding DINO (Swin B)	YOLOv8	+0.0027	+0.0031
	YOLO-NAS	−0.0388	−0.0215
Grounding DINO (Swin T)	YOLOv8	+0.0097	+0.0089
	YOLO-NAS	−0.0275	−0.0164
Detic	YOLOv8	+0.0509	+0.0416
OWL-ViT	YOLOv8	−0.0002	−0.0008
OWLv2	YOLOv8	+0.0492	+0.0393

**Table 3:** mAP50 and mAP50:95 metrics in MEP asset detection over 100 epochs.

		Asset			
Vision Language Model	Close-Set Object Detector	Valve		Pump	
		mAP50	mAP50:95	mAP50	mAP50:95
Grounding DINO (Swin B)	YOLOv8	0.928	0.889	0.778	0.662
	YOLO-NAS	0.8914	0.8207	0.7583	0.5779
	-(Fine-Tuned Model)	0.986	0.964	0.937	0.755
Grounding DINO (Swin T)	YOLOv8	0.875	0.813	0.653	0.523
	YOLO-NAS	0.8196	0.7336	0.6188	0.4478
GroundedSAM	YOLOv8	0.842	0.779	0.695	0.578
Detic	YOLOv8	0.612	0.492	0.439	0.295
OWL-ViT	YOLOv8	0.476	0.298	0.295	0.167
OWLv2	YOLOv8	0.465	0.282	0.503	0.27
-(Fully Supervised)	YOLO-NAS	0.9665	0.8216	0.9066	0.6815
-(Fully Supervised)	YOLOv8	0.9917	0.8837	0.93	0.734

**Table 4:** Comparative performance of vision language models combined with close-set object detectors for valve and pump detection in industrial scenes, evaluated using precision, recall, and F1-scores.

		Asset					
Vision Language Model	Close-Set Object Detector	Valve			Pump		
		Precision	Recall	F1-Score	Precision	Recall	F1-Score
Grounding DINO (Swin B)	YOLOv8	0.90	0.88	0.89	0.79	0.74	0.764
	YOLO-NAS	0.88	0.85	0.864	0.77	0.72	0.744
	-(Fine-Tuned Model)	0.97	0.96	0.965	0.93	0.91	0.92
Grounding DINO (Swin T)	YOLOv8	0.85	0.83	0.84	0.69	0.64	0.664
	YOLO-NAS	0.81	0.78	0.795	0.67	0.62	0.644
GroundedSAM	YOLOv8	0.82	0.80	0.81	0.73	0.70	0.715
Detic	YOLOv8	0.70	0.60	0.646	0.55	0.48	0.513
OWL-ViT	YOLOv8	0.55	0.50	0.524	0.40	0.35	0.373
OWLv2	YOLOv8	0.52	0.48	0.499	0.58	0.55	0.565
-(Fully Supervised)	YOLO-NAS	0.95	0.91	0.929	0.90	0.88	0.89
-(Fully Supervised)	YOLOv8	0.98	0.97	0.975	0.91	0.89	0.90

**Table 5:** Comparative analysis of computational efficiency among vision language models.

Vision Language Model	Average Inference Time (ms/Image)
OWL-ViT	65.9
Grounding DINO (Swin T)	88.7
Detic	216.5
Grounding DINO (Swin B)	234.4
GroundedSAM	251.4
OWLv2	301.9

## Data Availability

Some or all data, models, and code that support the findings of this study are available from the corresponding author upon request.
